# Non-invasive treatment of uterine fibroids using MR guided high intensity focused ultrasound: results on quality of life, non-perfused volume ratio and size reduction over 12 months

**DOI:** 10.1186/2050-5736-2-S1-A18

**Published:** 2014-12-10

**Authors:** Federica Ciolina, Fabrizio Boni, Fulvio Zaccagna, Luca Bertaccini, Beatrice Cavallo Marincola, Vincenzo Noce, Carola Palla, Alessandro Napoli, Carlo Catalano

**Affiliations:** 1 Department of Radiological Sciences, Oncology and Pathology, Sapienza University of Rome, Italy

## Purpose

To evaluate the efficacy of MR guided acoustic ablation in symptoms relief and volume reduction over time.

## Materials and method

110 leiomyomas in 98 women (average age 39,3 years) were treated with MRI-guided focused ultrasound (MRgFUS). The treatment is carried out using the ablative properties of the HIFU system under 3T MRI guide. Symptoms (e.g. menorrhagia, pelvic pain) were scored using Severity Score (SS) and quality of life was determined using the UFS-QOL score. Pre-treatment measurementes of leiomyoma volume were obtained by MR images. Immediately after treatment, Non-perfused Volume (NPV) was calculated from T1-w contrast-enhanced MR sequences. The average volume of treated fibroids was 90.27 ± 90.4 mm3. Follow-up images were obtained at 3 and 12 months after treatment and served to determine leiomyoma shrinkage. Qualitative and quantitative relations between fibroid volume, NPV ratio at treatment, and 12-month shrinkage were measured.

## Results

MRgFUS treated patients demonstrated a significant change in USF-QoL score: mean SS score values were 48.6 ± 13.4 (pre-treatment), 25.1 ± 8.9 (three-months follow-up) and 19,3± 6.8 (twelve-months follow-up). Fibroids volume changed from 90.27±90.4 mm3 (before treatment) to 54 ± 66.1 mm3 (at 12 months follow up). We encountered a statistically significant difference between the two values (p = 0.001). Mean post-treatment VNP was 57,65±52.9 mm3, about 63% of total fibroid volume (p=0.001).

## Conclusion

MRgFUS therapy of leyomioma results in a significant relief of symptoms and greater than 50% total fibroid ablation. The procedure is carried out in a totally non-invasive manner and features a high safety profile.

